# A Cadaveric Study of the Carpal Tunnel and Anatomical Variations of the Median Nerve in Vietnamese Adults: Implications for Single Portal Endoscopic Carpal Tunnel Release

**DOI:** 10.5704/MOJ.2203.002

**Published:** 2022-03

**Authors:** NT Ma, TD Tran, Q Tran, MC Duong

**Affiliations:** 1 Department of Experimental Surgery, Hanoi Medical University, Hanoi, Vietnam; 2 Department of Orthopaedic Surgery, College of Health Science VinUniversity, Hanoi, Vietnam; 3 Orthopaedic and Sports Medicine Center, Vinmec Healthcare System, Hanoi, Vietnam; 4 School of Population Health, University of New South Wales, Kensington, Australia

**Keywords:** carpal tunnel syndrome, transverse carpal ligament, endoscopic carpal tunnel release, superficial palmar arch, thenar motor branch

## Abstract

**Introduction:**

Single-portal endoscopic carpal tunnel release using modified Agee technique is widely used in Vietnam. Yet information on the anatomy of the target space of Vietnamese people regarding this technique is scarce. We aimed to characterise the anatomical landmarks and variations of the carpal tunnel to propose a safer surgery.

**Materials and methods:**

All twenty hands of ten fresh frozen, unembalmed cadavers of Vietnamese adults were included. Dissection was performed after the vertical line, Kaplan’s cardinal line and the distal wrist crease were drawn. The transverse carpal ligament (TCL), ulnar neurovascular bundle and superficial palmar arch were exposed. Measurements were made using Mitutoyo calliper. The variants of the median nerve and in the course of the thenar motor branch were recorded.

**Results:**

The median distances from the TCL distal margin to the distal wrist crease and superficial palmar arch were 31.2mm and 12.7mm, respectively. The ulnar neurovascular bundle was located 5.7mm and 4.4mm ulnar to the vertical line at the level of the TCL proximal margin and at the level of the TCL distal margin, respectively. The thenar motor branch of the median nerve was extra-ligamentous in 19 hands and preligamentous in 1 hand.

**Conclusion:**

If endoscopic portal is made along the distal wrist crease, blade assembly should not be inserted beyond the 35mm mark on its scale. Instruments should be aimed toward the radial border of the patient’s ring finger. Surgeons should be aware of the preligamentous course of the thenar motor branch although this variant type is rare.

## Introduction

Carpal tunnel syndrome (CTS), a condition caused by compression of the median nerve in carpal tunnel at the wrist, is the most common form of entrapment neuropathies^1^. The prevalence of CTS is approximately 3% of the general adult population^2^. The disorder is more common in women and the frequency increases with age^2^. Risk factors for CTS include obesity, jobs that require high hand/wrist repetition rate, forceful hand exertion, perimenopause, rheumatoid arthritis, use of hand-operated vibratory tools, and computer work^3^. The disorder is characterised by pain, paraesthesia, sensory deficit and motor impairment of the affected hand^1,2^. The treatment of CTS comprises non-surgical therapies and surgical intervention^4^. Non-surgical therapies, such as wrist splinting, steroid injection into the carpal tunnel, oral corticosteroids and physical therapy, are recommended to patients at an early stage of the disease^2,4^. However, these conservative treatments can only provide short-term improvement^3^. Surgical interventions, consisting of open surgery and endoscopic carpal tunnel release (ECTR) surgery that release the carpal tunnel by transection of the transverse carpal ligament (TCL), is the most radical treatment for patients with severe symptoms or if conservative treatments fail^1^. Of these surgical techniques, ECTR surgery is performed commonly due to its several advantages including less scarring and pillar pain as well as faster recovery time^4^. Although ECTR surgery is considerably a safe procedure, complications such as blood vessel and nerve damage and inadequate release of TCL may occur due to anatomic variations and preventable technical errors^4^. Common complications related to this technique include injuries to the ulnar neurovascular bundle, thenar motor branch (TMB), palmar cutaneous branch, and superficial palmar arch (SPA) as well as incomplete transection of the TCL^5^. To ensure a successful ECTR, a good understanding of the anatomic variations is recommended^4^. Single portal ECTR surgery using modified Agee technique for the transection of the TCL has been well described by Agee *et al* since 1992^6^ and has been demonstrated to be appropriate for Vietnamese people^7^. Based on our personal experience, single portal ECTR surgery using this technique has been increasingly implemented in Vietnam. However, there is no study on the anatomy of the target space in relation to single-portal ECTR technique. We conducted this study to characterise the anatomical landmarks and variations of the carpal tunnel to propose a safer carpal tunnel decompression for Vietnamese people and comparable populations.

## Materials and Methods

The study was conducted in May 2017 on hands of all fresh frozen cadavers of Vietnamese adults who donated their bodies to the Department of Anatomy at Pham Ngoc Thach University of Medicine in Vietnam for research and clinical training purposes. Exclusion criteria included having previous surgery, deformity or damage of the upper extremity, or history of musculoskeletal diseases, nervous diseases, or connective tissue disorders. All 20 hands of 10 fresh frozen cadavers were included in this study. The study was performed in accordance with the Declaration of Helsinki and was accepted by the Ethics Committee of Hanoi Medical University (reference No. 23/2017).

**Fig. 1:**
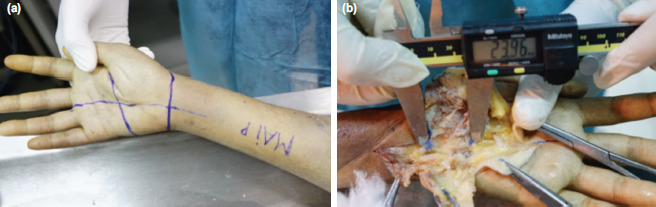
(a) The drawn lines. (b) Measuring the length of the transverse carpal ligament.

Ten fresh-frozen unembalmed cadavers were thawed at room temperature prior to dissection. After being thawed, a vertical line was drawn from the ulnar side of the palmaris longus to the radial border of the ring finger. In modified Agee technique, the blade assembly is inserted in line with this vertical line^8^. Kaplan’s cardinal line was subsequently drawn from the apex of the interdigital fold between the thumb and index finger to the hook of hamate. Finally, the distal wrist crease where the endoscopic portal is made in modified Agee technique was delineated (Fig 1a).

**Fig. 2:**
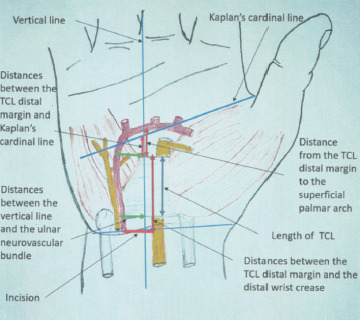
Orthopaedic measurements in single portal endoscopic carpal tunnel release.

**Fig. 3:**
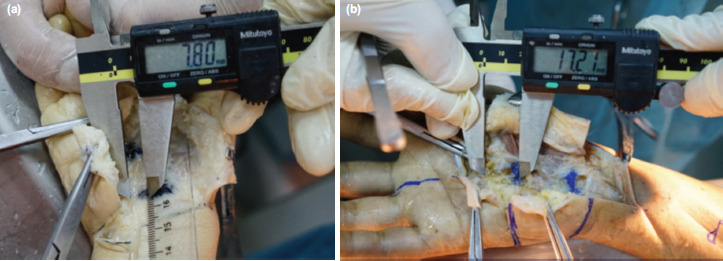
(a) Measuring distance between the vertical line and the ulnar neurovascular bundle. (b) Measuring distance between the distal margin of the transverse carpal ligament and the superficial palmar arch.

**Fig. 4:**
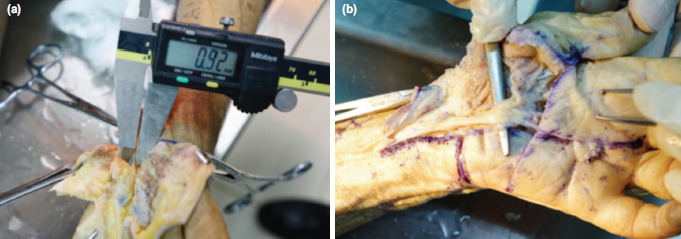
(a) Transverse carpal ligament thickness measurement. (b) Exposing the median nerve.

The SmartRelease® ESTR- a single-portal endoscopic surgery system [Micro-Aire Surgical Instruments, Charlottesville, Va, USA] was used^9^. An incision was made through the skin along the drawn lines on palmar side of each hand. Skin and subcutaneous tissue were dissected, and skin flaps were stripped away to reveal the palmar aponeurosis which was subsequently removed to expose the TCL. TCL was recognised by its transverse fibres which runs from the tubercle of the scaphoid bone and the trapezium bone on the radial side to the pisiform and the hook of the hamate bone on the ulnar side. The ulnar neurovascular bundle was then located upon the TCL on the ulnar border of wrist. The SPA was identified based on the direct continuity of the ulnar artery and the superficial palmar branch of the radial artery. The distances between (1) the TCL distal margin and the distal wrist crease, (2) the TCL distal margin and Kaplan’s cardinal line at the intersection of this line and the vertical line, and (3) the TCL distal margin and the SPA at the intersection of the arch and the vertical line were measured (Fig 2). Measurements of the distances between the vertical line and the ulnar neurovascular bundle at the levels of TCL proximal and distal margins were also made (Fig 3a). Subsequently, the length of TCL in the longitudinal direction was measured along the vertical line (Fig 1b). After measuring the length, the TCL was cut along the vertical line. The thickness of TCL was identified and measured (Fig 4a). The distance from the distal wrist crease to the SPA was calculated. It was the sum of the distance from the TCL distal margin to the distal wrist crease and the distance from the TCL distal margin to the SPA at the intersection of the arch (Fig 3b) and the vertical line (Fig 2). All measurements were performed by a single researcher (MNT) who is a qualified anatomist using a high precision digital caliper [Mitutoyo absolute 500 series, Mitutoyo Corp, Kawasaki, Japan] with an accuracy of ± 0.01 inch^10^.

The median nerve which is the only nerve passes through the carpal tunnel into the hand was exposed after cutting the TCL (Fig 4b). The anatomical variants of the median nerve including variable ramifications, bifurcation, and anastomosis between the median nerve and ulnar nerve were described. Variations in the course of the TMB of the median nerve were also recorded.

Data were managed and analysed using Statistical Package for the Social Sciences (SPSS) version 25 [SPSS Inc, Chicago, IL, United States]. The measurements were presented as median (lower quartile (LQ); upper quartile (UQ)) and range (minimum-maximum). The unit of measurements was mm. The frequency of cases with variants of the median nerve was also reported.

## Results

Twenty hands of six men and four women were included in the study. The median age of participants was 63.5 years (LQ; UQ 52; 84 years). The youngest participant was 25 years old and the oldest participant was 85 years old. The median distance from the TCL distal margin to the distal wrist crease was 31.2mm (30.4mm; 31.8mm) (Table I). The longest distance from the TCL distal margin to the distal wrist crease was 34.2mm. The median distance from the TCL distal margin to Kaplan’s cardinal line was 10.0mm (8.6mm; 10.7mm). The median distance between the TCL distal margin to SPA was 12.7mm (11.9mm; 13.6mm).

**Table I:** Distance from distal margin of the transverse carpal ligament to the drawn lines

Distance from the distal margin of the transverse carpal ligament (mm)	Mean (SD)	Median (LQ; UQ)	Range
To distal wrist crease	31.0 (1.9)	31.2 (30.4; 31.8)	26.0-34.2
To Kaplan’s cardinal line	10.0 (2.0)	10.0 (8.6; 10.7)	6.3-14.2
To superficial palmar arch	12.7 (2.5)	12.7 (11.9; 13.6)	7.6-17.2

The median distance from the distal wrist crease to the SPA was 44.2mm (41mm; 45.9mm). The shortest distance between them was 37.9mm (Table II). The median distance from the vertical line to the ulnar neurovascular bundle at the level of the TCL proximal margin was 5.7mm (5.0mm; 6.4mm). The shortest and longest distances between them were 4.3mm and 7.8mm, respectively. However, the ulnar neurovascular bundle was located closer to the vertical line at the level of the TCL distal margin (Fig 3a). The median distance between them at the level of the TCL distal margin was 4.4mm (4.2mm; 4.6mm). The median length of the TCL measured along the vertical line was 22.7mm (20.9mm; 24.6mm). The median thickness of the TCL was 2.8mm (2.6mm; 3.2mm) (Table III).

**Table II:** Distances between the distal wrist crease to the superficial palmar arch and between the vertical line to the ulnar neurovascular bundle

Distances (mm)		Mean (SD)	Median (LQ; UQ)	Range
From the distal wrist crease to the superficial palmar arch		43.6 (3.0)	44.2 (41; 45.9)	37.9-48.9
From the vertical line to	At the level of the TCL proximal margin	5.8 (1.0)	5.7 (5.0; 6.4)	4.3-7.8
the ulnar neurovascular bundle	At the level of the TCL distal margin	4.3 (0.9)	4.4 (4.2; 4.6)	2.5-6.1

**Table III:** Length and thickness of the transverse carpal ligament (TCL)

Measurements (mm)	Mean (SD)	Median (LQ; UQ)	Range
Length of the TCL	22.7 (3.0)	22.7 (20.9; 24.6)	16.4-28.4
Thickness of the TCL	2.9 (0.5)	2.8 (2.6; 3.2)	1.7-4.1

Anatomical variants of the median nerve were not detected in this study. However, a variant in the course of the TMB of the median nerve was recorded. The TMB of the median nerve was extra-ligamentous in 19 hands (95.0%) and preligamentous in only one hand (5.0%). Particularly, the preligamentous TMB was found only in the left hand of a 58year-old male cadaver. The TMB originated from the radial side of the main trunk of the median nerve 10.0mm proximal to the carpal tunnel, travelled through carpal tunnel and passed around the distal margin of the TCL to enter the muscles of the thumb.

## Discussion

In modified Agee technique, the endoscopic portal is made along the distal wrist crease and inserted in line with the vertical line that is drawn from the ulnar side of the palmaris longus to the radial border of the ring finger^8^. During insertion, the thumb of another hand of the surgeon should be placed over the safe zone, which is situated in line with the vertical line and proximal to Kaplan’s line, for perception of the movement of instruments^8^. When the instruments pass through the TCL distal margin, the thumb can feel their movement, and the insertion is ceased immediately. The TCL is incised by withdrawing the assembly. To release the TCL completely, the TCL division must be started from the TCL distal margin and the incision can be repeated if required^8^. The median distance from the TCL distal margin to the distal wrist crease was 31.2mm and ranged from 26.0-34.2mm. However, some cases (9/20 hands) had this distance of less than 31.2mm. There have not been anatomical landmarks which predict position of the TCL distal margin. Thus, perception of the movement of instruments by placing the thumb over the safe zone helps realise the instruments when they pass through the TCL distal margin. This palpation of the safe zone can ensure a complete release of the TCL and avoid the blade assembly inserted too deep in such cases.

The distance from the distal wrist crease to the SPA was calculated in order to estimate the distance that can be safe for the blade assembly insertion. Since the shortest distance between them was 37.9mm, introducing the blade assembly deeper than 37.9mm can cause injury to the SPA. In clinical practice, the smallest scale division of the blade assembly is 5mm. The longest distance from the distal wrist crease to the TCL distal margin recorded in this study was 34.2mm. As a result, for a safe and complete TCL release, it is unnecessary to insert the blade assembly beyond the 35mm mark shown on the scale. The median distance between the TCL distal margin and the SPA of 12.7mm in our study approximates the distances demonstrated by Samarakoon *et al* (11.48mm) and Omokawa *et al* (12mm)^11,12^. However, the distance that we measured is shorter than the result reported by Sacks *et al* (18.8mm), and longer than that of 5.5mm in a study conducted by Rotman *et al*^13,14^, which can be due to racial and ethnic differences^11,12,14^. We found that the Kaplan’s line was 10.0mm distal from the TCL distal margin. Meanwhile, the TCL distal margin and the SPA was in a median distance of 12.7mm. In other words, the SPA was just 2.7mm distal to the Kaplan’s line. This short distance was also demonstrated by Vella *et al*^15^. As a result, this line is used as a predictable landmark for the SPA^15^. Therefore, insertion of the instruments should be instantly ceased when their movement is felt by the thumb placed over the safe zone. A deeper insertion can cause damage to the SPA. The ulnar neurovascular bundle was located 5.7mm and 4.4mm ulnar to the vertical line at the level of the TCL proximal margin and at the level of the TCL distal margin, respectively. Consequently, the ulnar neurovascular bundle can get lacerated if the instruments are placed out of line with the vertical line and misaligned in ulnar direction. The median length of the TCL was 22.7mm in this study, which is markedly shorter than that of the TCL demonstrated by Samarakoon *et al* (27mm), Sacks *et al* (28.5mm) and Vasiliadis *et al* (31.0mm)^11,13,16^. This difference is probably due to the difference in the anthropometric measurements between races. We found that the median thickness of the TCL was 2.8mm and the maximum value was 3.4mm. Thus, the blade assembly should only be lifted 3mm during withdrawal. Then, the result of the transection of the TCL can be observed on a video screen placed in front of the surgeon during the surgery. If the TCL is just partially incised, the incision can be repeated to separate the TCL. The palmar aponeurosis should not be damaged when dividing the TCL.

Regarding variations in the course of the TMB of the median nerve, the extra-ligamentous TMB and the preligamentous TMB were recorded in 20 hands of Vietnamese cadavers, in which the extra-ligamentous type was most common (95%). A meta-analysis study also found that the extra-ligamentous TMB course was generally most common, but the prevalence rate of extra-ligamentous TMB course (75.2%) was lower than our result^17^. Indeed, the rate of extraligamentous TMB course in our study is higher than the rates reported by Sacks *et al* (92%), Al-Qattan *et al* (56%), Mizia *et al* (78.3%) and Samarakoon *et al* (84.6%)^13,18,19,11^. Despite being a very rare variant of the TMB of the median nerve, the preligamentous TMB course was documented in our study as well as in other studies in Brazil, Korea, India and Saudi Arabia^17,20-22^. Like the case reported in Brazil, the preligamentous TMB was only found on the left hand, not both hands of a cadaver. The preligamentous TMB in our case originated from the radial side of the median nerve – a common origin of the TMB^8,20^. Particularly, in our study, the preligamentous course was found on a male cadaver. This finding is similar to a case reported in Saudi Arabia^20^. Besides, we found that the TMB ran deep through the carpal tunnel, instead of travelling superficial to the TCL, to enter the muscles of thumb^17,20^. This rare variant of the TMB course increases the risk of injury to the TMB during the TCL release. Surgeons should be aware of the preligamentous TMB course before performing ECTR surgeries.

## Conclusion

In modified Agee technique, Kaplan’s line and the vertical line which are drawn from the ulnar side of the palmaris longus to the radial border of the ring finger should be delineated before performing skin incision. Endoscopic instruments should be inserted in line with the vertical line to avoid damage to the ulnar neurovascular bundle. During the introduction of the instruments, the surgeon’s thumb should be positioned over the safe zone to feel their movement. As the instruments pass through the TCL distal margin, the insertion should be instantly stopped to prevent the SPA from injury. For Vietnamese people, if the endoscopic portal is made along the distal wrist crease, the blade assembly should not be inserted beyond the 35mm mark shown on the scale of the blade assembly due to the risk of injury to the SPA. The transection of the TCL should be performed only when the entire length of the TCL is observed in order to achieve a complete release of the TCL. Extra-ligamentous and preligamentous courses are two variant types of the TMB course. Although the extra-ligamentous course is common, surgeons should always be aware of the preligamentous course before performing ECTR surgeries to reduce the risk of injury to the TMB. It is recommended that variations in the course of the TMB of the median nerve should be elaborated further in large-scale studies.
